# The risk factors and early predictive model of hematotoxicity after CD19 chimeric antigen receptor T cell therapy

**DOI:** 10.3389/fonc.2022.987965

**Published:** 2022-09-30

**Authors:** Yang Wang, Zhiqiang Song, Yuke Geng, Lei Gao, Lili Xu, Gusheng Tang, Xiong Ni, Li Chen, Jie Chen, Tao Wang, Weijia Fu, Dongge Feng, Xuejun Yu, Libing Wang, Jianmin Yang

**Affiliations:** ^1^ Department of Hematology, Institute of Hematology, Changhai Hospital, Second Military Medical University, Shanghai, China; ^2^ HuaDao Biopharma (Shanghai) Limited Corporation, Shanghai, China

**Keywords:** risk factors, early predictive model, hematotoxicity, chimeric antigen receptor T cell, acute lymphoblastic leukemia, large B-cell lymphoma

## Abstract

Hematotoxicity is the most common long-term adverse event after chimeric antigen receptor T cell (CAR-T) therapy. Here, a total of 71 patients with relapsed or refractory (R/R) B-cell acute lymphoblastic leukemia (B-ALL) or large B-cell lymphoma (LBCL) were used to develop an early hematotoxicity predictive model and verify the accuracy of this model. The incidences of early hematotoxicity at 3 month following CAR-T infusion in B-ALL and LBCL were 45.5% and 38.5%, respectively. Multivariate analyses revealed that the severity of cytokine release syndrome (CRS) was an independent risk factor affecting early hematotoxicity. The analysis between the peak cytokine levels and early hematotoxicity suggested that tumor necrosis factor-α (TNF-α) and C-reactive protein (CRP) were closely associated with early hematotoxicity. Then, an early predictive model of hematotoxicity was constructed based on the peak contents of TNF-α and CRP. This model could diagnose early hematotoxicity with positive predictive values of 87.7% and 85.0% in training and validation cohorts, respectively. Lastly, we constructed the nomogram for clinical practice to predict the risk of early hematotoxicity, which performed well compared with the observed probability. This early predictive model is instrumental in the risk stratification of CAR-T recipients with hematotoxicity and early intervention for high-risk patients.

## 1 Introduction

Many clinical trials have demonstrated that CD19 chimeric antigen receptor T cell (CAR-T) therapy is a promising treatment for patients with relapsed or refractory (R/R) hematological malignancies (1–3). The complete response (CR) rates following CAR-T therapy in patients with B-cell acute lymphoblastic leukemia (B-ALL) (4), chronic lymphocytic leukemia (5), and large B-cell lymphoma (LBCL) (6) were 68–93%, 21%, and 40–59%, respectively. Up to now, the Food and Drug Administration has approved four CD19 CAR-T products for treating relapsed or refractory (R/R) hematological malignancies; axicabtagene-ciloleucel (Yescarta), tisagenlecleucel (Kymriah), brexucabtagene autoleucel (Tecartus), and lisocabtagene maraleucel (Breyanzi). Furthermore, other targets and multiple target CAR-T products have also been widely studied in clinical trials (7–9).

Despite their remarkable clinical efficacy, further refinement of CAR-T therapy-associated adverse events is imperative, including cytokine release syndrome (CRS) (10), immune effector cell-associated neurotoxicity syndrome (ICANS) (11), and hematotoxicity (12). It has previously been reported that hematotoxicity is a long-term and serious side effect of CAR-T therapy (13, 14), potentially causing anemia, hemorrhagic events, and resulting in a higher incidence of infection. Abramson et al. found that 37% of evaluable patients who received CAR-T therapy developed grade 3 cytopenia at 1-month post-treatment (15). It was also reported that 48% of patients had grade 3-4 cytopenia on day 30 after CAR-T infusion, including anemia, neutropenia, or thrombocytopenia (16). Ultimately, hematotoxicity increases the treatment burden, and leads to higher mortality rates. Thus, it is of great clinical importance to identify the independent risk factors of early hematotoxicity to reduce the probability of hematotoxicity and improve hematopoietic function.

Although the toxicity and risk factors of CRS and ICANS have been well studied and reported (10, 17), there is limited understanding of early hematotoxicity after CAR-T therapy. Several retrospective studies have attempted to identify the risk factors associated with hematotoxicity. Jain et al. found that serious CRS and ICANS were significantly associated with absence of blood complete recovery (BR) (18). Additionally, Juluri et al. reported that higher serum TGF-β levels were positively correlated with BR, while increased IL-6 levels were negatively correlated with BR (19). However, the characteristics and risk factors of early hematotoxicity remain unclear, and further research is required to develop an early predictive model of hematotoxicity.

In this study, we collected and analyzed the possible clinical factors affecting early hematological toxicity (3 month after CAR-T infusion) in patients with hematological malignancy who received CD19 CAR-T therapy. We observed a positive correlation between the severity of CRS and early hematotoxicity, and developed an early predictive model based on peak levels of tumor necrosis factor-α (TNF-α) and C-reactive protein (CRP) during the first week following CAR-T therapy. This early predictive model of hematological toxicity performed well in the training and validation cohorts. Lastly, we constructed the nomogram for clinical practice to predict the risk of early hematotoxicity.

## 2 Materials and methods

### 2.1 Patients and study design

From December 2015 to March 2022, 47 patients with R/R B-ALL and 44 patients with R/R LBCL received CD19 CAR-T therapy at Changhai Hospital, the First Affiliated Hospital of the Second Military Medical University (Shanghai, China). The inclusion criteria for this study were: (1) CD19^+^ B-ALL or LBCL diagnosed using histology and immunohistochemical evaluation according to the 2008 WHO classification of hematological malignancies; (2) refractory or relapsed disease according to the National Comprehensive Cancer Network Guidelines; (3) one month medical records following CAR-T therapy were complete and accessible; and (4) patients who received only one CAR-T treatment and did not present complications with other malignancies. Additionally, 20 patients were excluded because of death within 3 month after CAR-T cell infusion or incomplete data. Among them, 8 patients died due to disease progression, 3 died due to neutropenic infection, 1 died due to intracranial hemorrhage and 8 were excluded due to incomplete data of hematopoietic recovery. A total of 22 R/R B-ALL and 13 R/R LBCL patients were enrolled in training cohort to construct the early predictive model of hematopoietic recovery. Finally, a validation cohort (9 R/R B-ALL and 27 R/R LBCL) was used to verify the accuracy of predictive model. Written informed consent was obtained before CAR-T therapy, and this clinical trial (ChiCTR-OIN-15007668) was approved by the institutional review board of Changhai Hospital.

### 2.2 CAR-T products and lymphodepletion treatment

CAR-T cells were produced by Shanghai HuaDao Biopharma Limited Corporation. First, leukapheresis was used to collect T cells from peripheral blood mononuclear cells (PBMCs). Subsequently, T cells were activated using CD3 (1000 µg/mL) and CD28 (500 µg/ml) microbeads. Activated T cells were then transduced with a lentiviral vector encoding T cell chimeric antigen receptor, composed of a CD19-specific single-chain variable fragment, hinge domain, transmembrane domain, and CAR-T costimulatory domain of 4-1BB and CD3 ζ. Briefly, interleukin (IL)-2 (600 IU/mL) was applied to expand the T cells. Finally, CAR-T cells were infused into patients after a series of laboratory tests and sample processing (20, 21). In addition, fludarabine and cyclophosphamide were used as lymphodepletion chemotherapy before CAR-T cell infusion (22).

### 2.3 Definitions

The criteria for haematological “recovery” at 3 month were defined according to a previously published study (18). Briefly, these parameters included: white blood cell (WBC) count > 3 × 10^9^/L and neutrophil counts > 1 × 10^9^/L without growth factor administration in two weeks; platelets > 50 × 10^9^/L without transfusions in one week and hemoglobin > 8 g/dL in the absence of red cell transfusion in two weeks. Baseline blood counts were the hematological counts before lymphodepletion chemotherapy. The “normalization” of hematological counts were defined as follow: WBC ≥ 4 × 10^9^/L, neutrophil ≥ 2 × 10^9^/L, platelets ≥ 100 × 10^9^/L and hemoglobin ≥ 11 g/dL for women or 12 g/dL for men. “Blood complete recovery (BR)” was defined as the recovery of all the four cell counts mentioned above, and other situations were recognized as Non-BR.

CRS was graded based on the American Society of Transplantation and Cellular Therapy (ASTCT) (23). The severity of cytopenia was defined according to the Common Terminology Criteria of Adverse Events (CTCAE, v5.0), and severe cytopenia was defined as grade ≥3 anemia, neutropenia, and thrombocytopenia. In addition, cytokine analysis was performed according to the results of the laboratory tests in Changhai Hospital. The cytokines analyzed in this study included TNF-α, IL-10, IL-2R, IL-6, IL-8, and CRP. In addition, progression-free survival (PFS) was defined as the time from CAR-T infusion to disease progression. Overall survival (OS) was defined as the time from CAR-T infusion to death due to any cause or the last follow-up.

### 2.4 Risk factors and early predictive model of hematopoietic recovery

Firstly, univariate analysis between BR and Non-BR groups was performed among multiple variables, including age, gender, disease type, baseline tumor burden, lines of prior therapies, baseline blood cell count, CAR-T cell dose, and CRS grade. The variables with P < 0.05 were identified as significantly different factors associated with hematopoietic recovery. Previous studies have proved that baseline blood counts were closely related to hematopoietic recovery following CAR-T therapy (18, 19). Therefore, significantly different factors and baseline blood counts were included to perform multivariate logistic regression analyses, so as to identify independent risk factors associated with BR. Finally, the severity of CRS was recognized as the independent risk factor related to hematopoietic recovery.

To construct an early predictive model of hematopoietic recovery, the relationship between peak cytokine levels during 1 week after CAR-T therapy and BR was analyzed. Finally, only the cytokines with P < 0.1 between the BR and Non-BR groups, and area under the curve (AUC) > 0.6 were identified as predictive biomarkers (24).

### 2.5 Statistical analysis

Statistical software (SPSS 22.0, SPSS Inc., Chicago, IL, USA) and GraphPad Prism 7.00 software (GraphPad Software, La Jolla, CA, USA) were used to analyze statistical data and create figures. The comparison between subgroups was performed by using Fisher’s exact test for categorical variables and t-test for continuous variables. Kaplan–Meier curves were used to analyze the survival possibility of PFS and OS, and survival differences were determined using the log-rank test. Univariate and multivariate analyses were performed to identify independent risk factors associated with early hematotoxicity. All P values reported were two-sided, and P values lower than 0.05 were considered significant. The nomogram was performed using R (version 3.6.3), RStudio (version 1.4.1106), and the following R packages: survival, survminer, survfit, ggsurvplot, and givitiR.

## 3 Results

### 3.1 Patient characteristics

**Figure 1:**
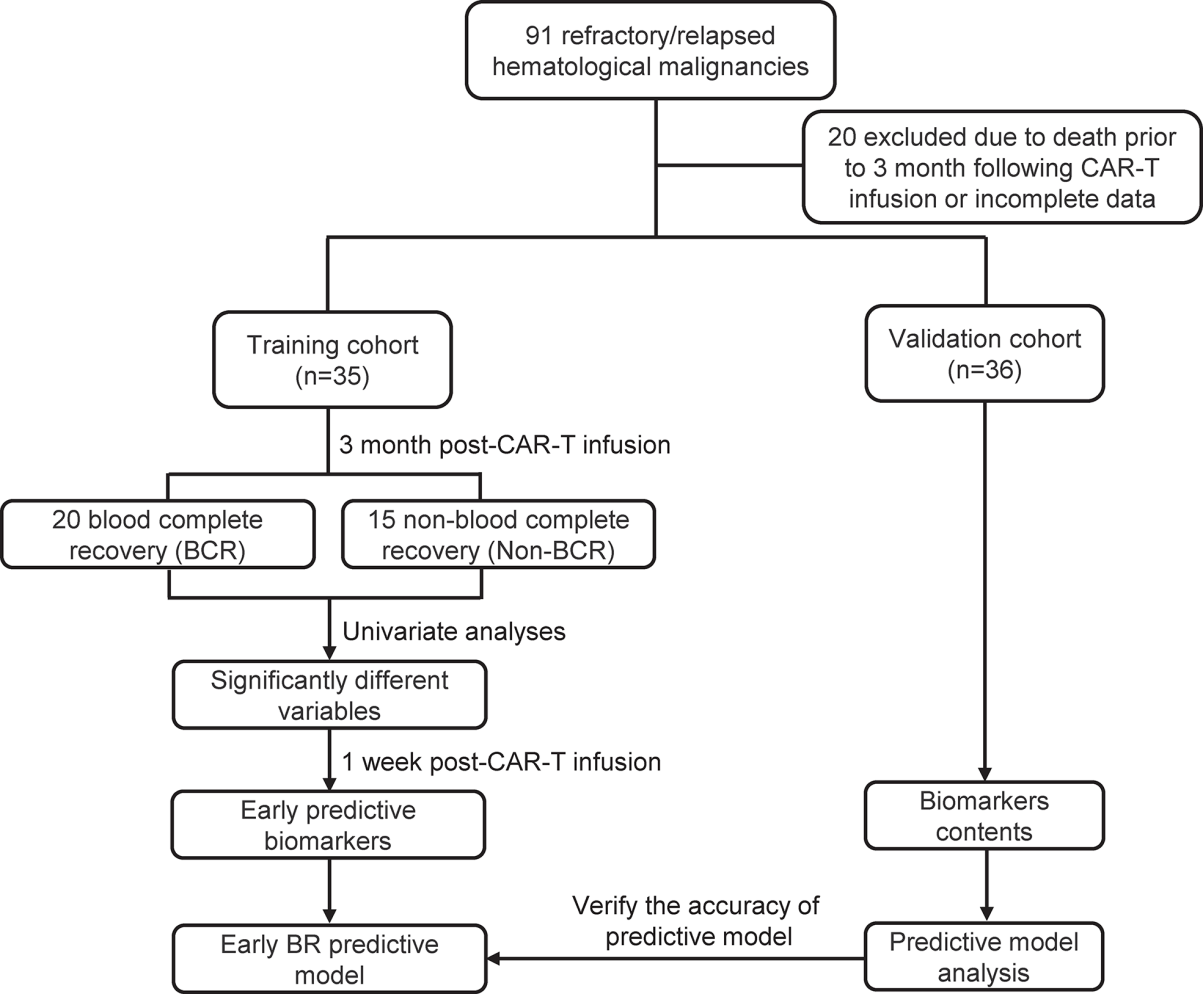
The flowchart of this study.

The flowchart of this study was presented in **Figure 1**. As shown in **Figure 1** and **Table 1**, a total of 35 patients with R/R B-cell hematological malignancies were enrolled in training cohort, comprising 22 (62.9%) R/R B-ALL and 13 (37.1%) R/R LBCL patients in this study. LBCL patients included 11 diffuse large B-cell lymphomas, 1 transformed mantle cell lymphoma, and 1 transformed Hodgkin lymphoma patients. The number of male and female patients was nearly equal, with a median age of 43 (range, 12–66) years. Of note, the median bone marrow blasts prior to CAR-T therapy was 8% (range, 0–92) in B-ALL patients, and only two LBCL patients had bone marrow invasion before CAR-T cell infusion. In patients with large B-cell lymphoma, the median baseline lactate dehydrogenase (LDH) contents was 263 U/L (range:173-1404). Moreover, two patients suffered from immune effector cell-associated neurotoxicity syndrome (ICANS) following CAR-T infusion.

**Table 1:** Patient and disease characteristics.

Variables	Training cohort	Validation cohort
**Age, median (range)**	43 (12-66)	43 (19-69)
**Gender, n (%)**		
Male	18 (51.4)	20 (55.6)
Female	17 (48.6)	16 (44.4)
**Disease, n (%)**		
B-ALL	22 (62.9)	9 (25)
Large B-cell lymphoma	13 (37.1)	27 (75)
**Tumor burden prior CAR-T**		
**B-ALL**		
Bone marrow blasts, median (%, range)	8 (0-92)	26.8 (0-93)
**Large B-cell lymphoma**		
**Ann Arbor stage**		
I-II	2 (15.4)	4 (14.8)
III-IV	11 (84.6)	23 (85.2)
**IPI score**		
0-2	7 (53.8)	14 (51.9)
3-5	6 (46.2)	13 (48.1)
**LDH levels pre-lymphodepletion, median (range)**	263 (173-1404)	228 (113-1289)
**Lines of prior therapies, median (range)**	6 (2-18)	7 (4-16)
**Baseline blood count, median (range)**		
WBC (×10^9^/L)	4.82 (0.13-22.61)	3.7 (1.44-13.03)
Neutrophil (×10^9^/L)	2.92 (0.06-14.11)	2.49 (0.82-11.91)
Hemoglobin (g/dL)	10 (4.8-14.6)	10.5 (5.8-13.2)
Platelet (×10^9^/L)	140 (14-294)	168 (54-401)
**Prior autologous HSCT, n (%)**	9 (25.7)	12 (33.3)
**Prior allogeneic HSCT, n (%)**	7 (20)	10 (27.8)
**CAR-T cell dose (×10^6^/kg)**	2.9 (0.35-6.7)	3 (0.1-11.76)
**CRS grade, n (%)**		
0	5 (14.3)	4 (11.1)
1	14 (40)	20 (55.6)
2	8 (22.9)	5 (13.9)
3	5 (14.3)	3 (8.3)
4	3 (8.5)	4 (11.1)
**ICANS, n (%)**	2 (5.7)	2 (5.6)
**3 month blood count after CAR-T cell therapy, median (range)**		
WBC (×10^9^/L)	3.85 (0.02-11.52)	3.91 (0.17-6.78)
Neutrophil (×10^9^/L)	2 (0.01-5.31)	1.77 (0.1-3.8)
Hemoglobin (g/dL)	10.3 (3.1-15.8)	12.3 (6.1-15.8)
Platelet (×10^9^/L)	90 (3-242)	154 (9-283)
**Blood complete recovery, n (%)**	20 (57.1)	26 (72.2)

B-ALL, B-cell acute lymphoblastic leukemia; LDH, lactate dehydrogenase; IPI, international prognostic index; CRS, cytokine release syndrome; HSCT, hematopoietic stem cell transplantation; WBC, white blood cell; CAR-T, chimeric antigen receptor T cell; ICANS, immune effector cell-associated neurotoxicity syndrome.

Meanwhile, the median line of prior therapy before CAR-T cell infusion was 6 (range, 2–18). 9 (25.7%) patients received autologous hematological stem cell transplantation (HSCT), and 7 (20%) patients received allogeneic HSCT before CAR-T therapy. The median CAR-T cell infusion dosage was 2.9 × 10^6/^kg (range, 0.35-6.7). Meanwhile, the median WBC, neutrophil, hemoglobin, and platelet counts before lymphodepletion (baseline blood count) were 4.82 × 10^9^/L, 2.92 × 10^9^/L, 10 g/dL and 140 × 10^9^/L, respectively, and 3 month post-CAR-T therapy blood counts were 3.85 × 10^9^/L, 2 × 10^9^/L, 10.3 g/dL and 90 × 10^9^/L, respectively. Furthermore, 20 (57.1%) patients showed blood complete recovery (BR) at 3 month after CAR-T cell infusion. Additionally, the patients characteristics of validation cohort were also presented in **Table 1**, including 9 R/R B-ALL and 27 R/R LBCL.

### 3.2 Treatment response and hematological recovery

**Figure 2:**
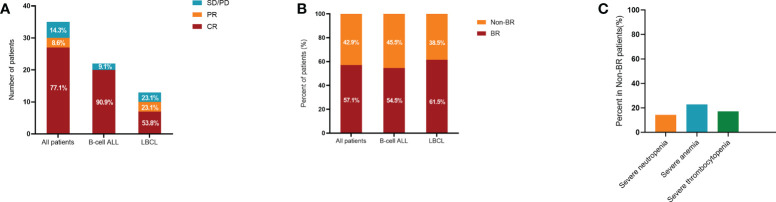
Treatment response and hematological recovery in the training cohort after CAR-T therapy. Treatment response **(A)** and hematological recovery **(B)** in patients with B-ALL and LBCL. Compared to patients with B-ALL, lower CR rate while higher BR rate were observed in patients with LBCL. **(C)** Incidences of severe neutropenia, anemia, and thrombocytopenia in Non-BR patients. CAR-T, chimeric antigen receptor T cell; B-ALL, B-cell acute lymphoblastic leukemia; LBCL, large B-cell lymphoma; CR, complete response; BR, blood complete recovery; Non-BR, non-blood complete recovery.

The median follow-up of this study was 9 months (range: 3-70). In the training cohort, 14 patients (40%) developed grade 1 cytokine release syndrome (CRS) and 16 patients (45.7%) experienced grade 2–4 CRS after CAR-T cell infusion. In particular, 5 patients (14.3%) had no obvious CRS (**Table 1**). The overall response rate (ORR) of all 35 R/R patients was 85.7%, and the rates of ORR in B-ALL and LBCL were 90.9% and 76.9%, respectively (**Figure 2A**). In addition, CR rates were 90.9% and 53.8% in B-ALL and LBCL patients, respectively (**Figure 2A**). Of note, 23.1% of patients with LBCLsuffered progressive disease after CAR-T cell infusion. In the validation cohort, 12 patients (33.3%) suffered from the severe CRS (grade ≥ 2) and 2 patients (5.6%) were reported to have ICANS (**Table 1**). Additionally, 26 patients (72.2%) had BR at 3 month after CAR-T therapy.

**Figure 3:**
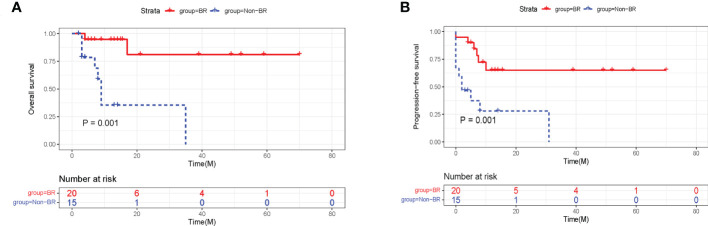
The effects of hematopoietic recovery on PFS and OS probability in training cohort. The survival analysis revealed that OS **(A)** and PFS **(B)** were significantly superior in patients with BR. PFS, progression-free survival; OS, overall survival; BR, blood complete recovery; Non-BR, non-blood complete recovery.

In the training cohort, 57.1% of patients were evaluated as having BR at 3 month after CAR-T therapy, while 42.9% of patients did not achieve BR (Non-BR). The BR rates in ALL and lymphoma were 54.5% and 61.5%, respectively (**Figure 2B**). In particular, the rates of severe neutropenia, anemia, and thrombocytopenia were 14.3%, 22.9%, and 17.1%, respectively (**Figure 2C**). Furthermore, patients with BR had notably longer PFS and OS compared to patients without BR (P < 0.05, **Figures 3A, B**). The median PFS and OS in patients with Non-BR were 2 and 9 months, respectively. Meanwhile, the median PFS and OS was not reached in patients with BR. Therefore, early hematopoietic recovery is important in patients with hematological malignancies undergoing CAR-T therapy.

### 3.3 Independent clinical factors affecting hematopoietic recovery

To identify the risk factors that potentially affect the early BR at 3 month after CAR-T therapy, all patients of training cohort were divided into BR and Non-BR subgroups according to their characteristics (**Table 2**). We collected and compared the data of age, sex, disease categories, baseline tumor burdens, lines of prior therapies, baseline blood counts, prior autologous or allogeneic HSCT, CAR-T cell dose, CRS grade, and 3 month post-CAR-T therapy blood counts in the two subgroups. The results indicated that the BR rate was significantly higher in patients with a low CRS grade (grade 0–1) (**Table 2**). None of the other characteristics were notably correlated with BR (P > 0.05). Additionally, previous studies have proved that baseline blood cell counts were closely related to the hematopoietic recovery (18, 19). Therefore, baseline blood cell counts (WBC, hemoglobin, and platelet) and CRS grade were included in multivariate logistic regression analyses. Finally, 4 variables (baseline WBC, hemoglobin, and platelet counts and CRS grade) were included into multivariate logistical analyses and the sample size of training cohort was 35, which met the requirement of statistical requirement (25). Our finding revealed that the severity of CRS independently affected BR (P < 0.05, **Supplementary Table S1**). And we concluded that CRS severity (grade ≥ 2) was an independent risk factor influencing early hematopoietic recovery, which was consistent with previous studies (19, 26). Although the PFS between the high and low CRS grades was not significantly different (P > 0.05, **Supplementary Figure 1A**), the OS in patients with low CRS grade was significantly longer than in those with high CRS grade (P < 0.05, **Supplementary Figure 1B**).

**Table 2:** Subgroup analysis of BR at 3 month after CAR-T cell infusion in training cohort.

Variables	BR (n=20)	Non-BR (n=15)	P value
**Age, median (range)**	48 (12-64)	36 (17-66)	0.173
**Gender, n (%)**			0.851
Male	10 (50)	8 (53.3)	
Female	10 (50)	7 (46.7)	
**Disease, n (%)**			0.583
B-ALL	12 (60)	10 (66.7)	
Large B-cell lymphoma	8 (40)	5 (33.3)	
**Tumor burden prior CAR-T**			
**B-ALL**			
Bone marrow blasts, median (%, range)	4.5 (0-61)	11.8 (0-92)	0.396
**Large B-cell lymphoma**			
**Ann Arbor stage**			0.487
I-II	2 (10)	0	
III-IV	6 (30)	5 (33.3)	
**IPI score**			1.000
0-2	4 (20)	3 (20)	
3-5	4 (20)	2 (13.3)	
**LDH levels pre-lymphodepletion (U/L), median (range)**	242 (173-891)	372 (263-1404)	0.246
**Lines of prior therapies, median (range)**	6 (2-18)	7 (3-15)	0.755
**Baseline blood count, median (range)**			
WBC (×10^9^/L)	4.75 (0.13-22.61)	4.74 (1.89-15.15)	0.242
Neutrophil (×10^9^/L)	3.02 (0.06-14.11)	2.6 (1.37-10.33)	0.443
Hemoglobin (g/dL)	10.7 (6.4-12.6)	8.9 (4.8-14.6)	0.121
Platelet (×10^9^/L)	142 (58-294)	133 (14-265)	0.091
**Prior HSCT, n (%)**	11 (55)	5 (33.3)	0.214
Autologous, n (%)	6 (30)	3 (20)	
Allogeneic, n (%)	5 (25)	2 (13.3)	
**CAR-T cell dose (×10^6^/kg), median (range)**	2.59 (0.35-6.7)	3.1 (0.87-4)	0.287
**CRS grade, n (%)**			0.031
0-1	14 (70)	5 (33.3)	
2-4	6 (30)	10 (66.7)	
**3 month blood count after CAR-T cell therapy, median (range)**			
WBC (×10^9^/L)	4.57 (3.1-11.52)	3.53 (0.02-7.43)	0.017
Neutrophil (×10^9^/L)	2.18 (1.18-5.31)	1.55 (0.01-3.4)	0.014
Hemoglobin (g/dL)	11.1 (8.8-14.9)	7.8 (3.1-15.8)	0.002
Platelet (×10^9^/L)	144 (51-242)	59 (3-203)	0.010

BR, blood complete recovery; LDH, lactate dehydrogenase; IPI, international prognostic index; CRS, cytokine release syndrome; HSCT, hematopoietic stem cell transplantation; WBC, white blood cell; CAR-T, chimeric antigen receptor T cell.

Baseline blood counts were numerically higher in patients with BR than in those without BR. However, none of them were significantly different (P > 0.05, **Table 2**). In particular, we observed that bone marrow blasts prior to CAR-T therapy in BR group were numerically lower than those in Non-BR group in patients with B-ALL, but the difference was not significant (P = 0.396, **Table 2**). Moreover, the differences of baseline tumor burdens (Ann Arbor stage, IPI score, and LDH levels) in patients with LBCL were not significant between BR and Non-BR groups.

### 3.4 Cytokines associated with hematopoietic recovery

**Figure 4:**
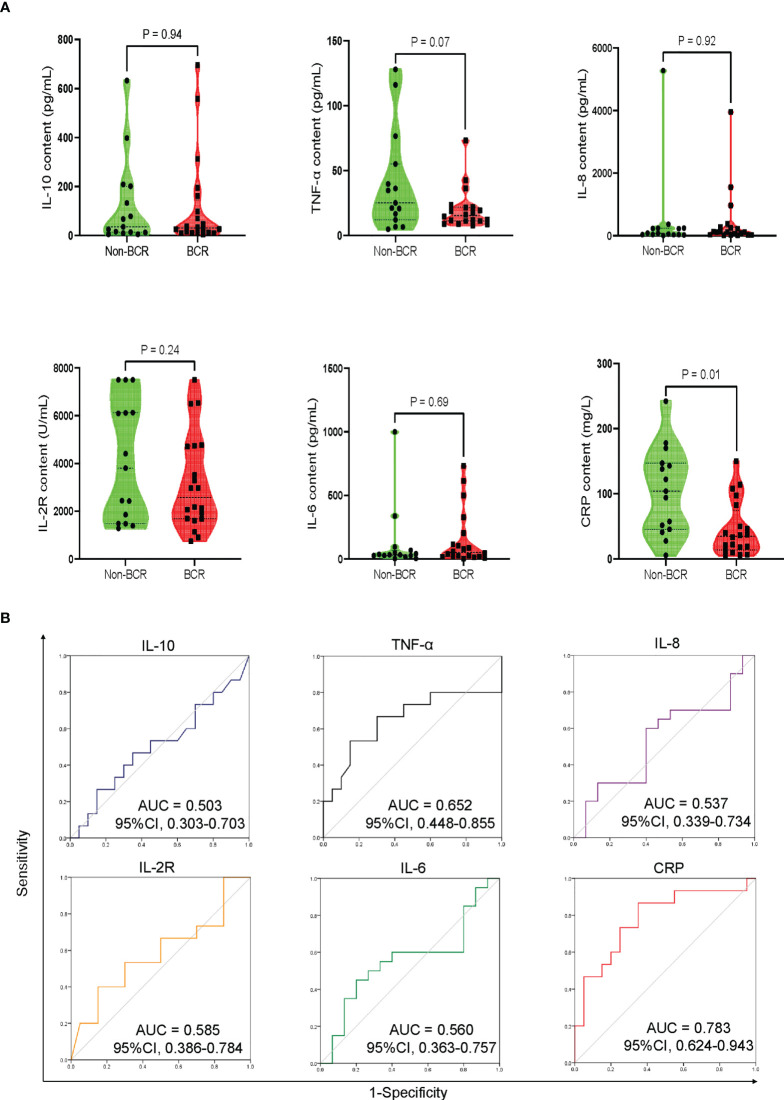
Peak levels and discriminative power of CRS-related cytokines in BR and Non-BR subgroups of training cohort. **(A)** The peak levels of CRP was significantly lower in BR patients compared to that in Non-BR patients. **(B)** Discriminative ability of cytokines between BR and Non-BR subgroups. The peak levels of TNF-α and CRP represented good discriminative power with AUC > 0.6. CRS, cytokine release syndrome; BR, blood complete recovery; Non-BR, non-blood complete recovery; CRP, C-reactive protein; TNF-α, tumor necrosis factor-α.

The results indicated that the severity of CRS was an independent risk factor closely related to early BR at 3 month after CAR-T cell therapy (**Supplementary Table S1**). Because most patients developed CRS at 1 week after CAR-T infusion, we presumed that CRS-related cytokines were correlated with early hematopoietic recovery and could predict the possibility of BR or Non-BR earlier than the clinical manifestation. Therefore, we investigated the peak levels of various cytokines at 1 week following CAR-T infusion, including TNF-α, IL-10, IL-2R, IL-6, IL-8, and CRP. The results indicated that the peak CRP levels were significantly lower in patients with BR than in those without BR (**Figure 4A**). Notably, the peak level of TNF-α was numerically higher in Non-BR group than in the BR group (P = 0.07, **Figure 4A**). The median time of peak levels of TNF-α and CRP was 3 days (range: 1-6) following CAR-T therapy. Meanwhile, there was no significant difference in the peak levels of IL-10, IL-2R, IL-6, and IL-8 between the BR and Non-BR subgroups (P > 0.05, **Figure 4A**).

Further, receiver operating characteristic (ROC) curves were generated to analyze the discriminative power of cytokines between the BR and Non-BR subgroups. The peak levels of TNF-α and CRP showed good discriminative ability with an area under the curve (AUC) > 0.6 (**Figure 4B**). Based on the criteria of P < 0.1 between the BR and Non-BR groups, and AUC > 0.6 (24), we believed that the peak levels of TNF-α and CRP during 1 week post-CAR-T therapy were closely associated with early hematopoietic recovery.

### 3.5 Construction and validation of early predictive model of hematopoietic recovery

**Figure 5:**
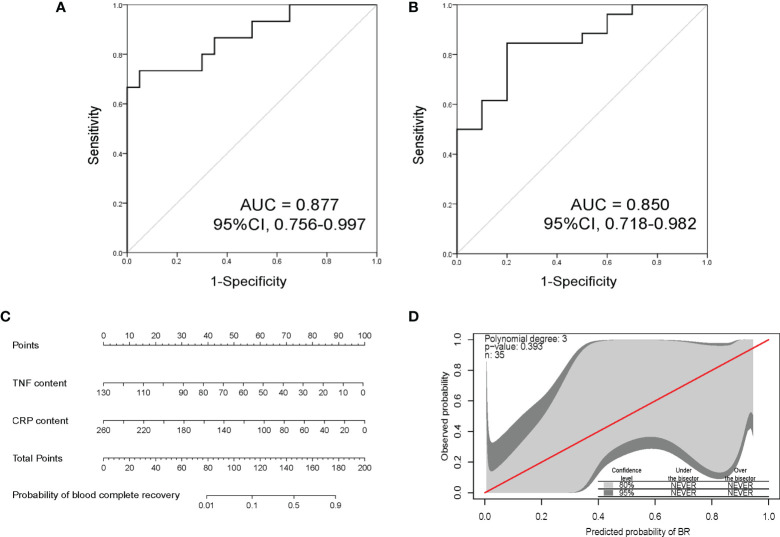
The discrimination power and nomogram of early predictive model. **(A)** Receiver operating characteristic analysis of this early BR predictive model in THE training cohort. This model could early predict hematotoxicity with AUC = 0.877. **(B)** Validation of this early BR predictive model in an independent cohort. This predictive model showed good accuracy with AUC = 0.850. **(C)** Nomogram based on the peak contents of TNF-α and CRP to evaluate the possibility of BR. **(D)** Early predictive model calibration curves comparing predicted probability of BR with observed early BR. The red line is perfect calibration line between the predicted probability and observed. The calibration belts of light gray and dark gray represent the 80% and 95% confidence level of this early predictive model, respectively. The model indicates good fit if calibration belts include the red line and P value > 0.05. BR, blood complete recovery; AUC, area under the curve; CRP, C-reactive protein; TNF-α, tumor necrosis factor-α.

According to the selective criteria factors with P < 0.1 between BR and Non-BR groups, concomitant AUC > 0.6 were identified as potential biomarkers. We thus selected the peak levels of TNF-α and CRP as predictive biomarkers of early BR. Based on the peak contents of TNF-α and CRP during 1 week after CAR-T therapy, we developed a predictive model of early hematopoietic recovery to analyze the possibility of BR. Moreover, we further validated this predictive model in an independent cohort. This model could diagnose early hematotoxicity with positive predictive value of 87.7% and 85.0% in training and validation cohorts, respectively (**Figures 5A, B**). Then, in order to help predict the risk of hematological toxicity of every individual in clinical practice, we constructed an BR evaluation nomogram could help predict the possibility of BR in each individual in clinical practice (**Figure 5C**). Lastly, GiViTi calibration belt of this early predictive model indicated good agreement between predicted possibility of BR and the observed early BR (P = 0.393, **Figure 5D**).

## 4 Discussion

In this study, we analyzed the characteristics of patients to identify the independent risk factors affecting early hematotoxicity. Combined with univariate and multivariate analyses, the severity of CRS was recognized as an independent clinical factor associated with early hematopoietic recovery. In addition, to construct an early predictive mode of hematopoietic recovery, we analyzed the peak cytokine levels at 1 week after CAR-T infusion, and TNF-α and CRP were identified as predictive biomarkers related to hematopoietic recovery. Finally, we developed an early predictive model to analyze the possibility of BR based on the peak contents of TNF-α and CRP and validated this model in an independent cohort. This model is instrumental in risk stratification of patients with hematotoxicity and individualized treatment for prevention of hematotoxicity in high-risk patients. Of note, although we developed this predictive model according to the peak levels of TNF-α and CRP at 1 week following CAR-T therapy, it is still applicable at any time within 1 week post-CAR-T infusion, even before the occurrence of CRS.

Hematotoxicity is a major adverse event in patients with hematological malignant tumors following CAR-T therapy, and approximately 38% of patients had grade 3 thrombocytopenia at 3 months after treatment, and ZUMA-1 reported that 17% of patients had grade ≥ 3 cytopenia (6, 27). However, the underlying mechanisms and clinical factors affecting early hematotoxicity remain unclear. Therefore, we performed this study to identify independent risk factors associated with early hematotoxicity and to construct an early predictive model to analyze the possibility of early BR.

In this retrospective analysis, we found that age, sex, disease type, baseline tumor burdens, lines of prior therapy, baseline blood counts, CAR-T cell dose, and prior autologous or allogeneic HSCT were not significantly different between the BR and Non-BR subgroups (**Table 2**). Other published studies have also reported that age and sex were not risk factors affecting hematotoxicity after CAR-T cell treatment (18, 19), which was consistent with our results. BR rates were numerically lower in patients with B-ALL than in those with LBCL (54.5% vs. 61.5%, **Figure 2B**). This may be associated with high bone marrow tumor burden in B-ALL, while only two patients had bone marrow invasion in LBCL. Previous lines of therapy before CAR-T infusion were not related to the treatment response (13, 28). Similarly, we found that the number of prior therapies was not associated with early hematopoietic recovery.

Juluri’s research proved that pre-lymphodepletion hemoglobin and neutrophil counts were not predictors of hematopoietic recovery following CAR-T cell infusion (19). Fried et al. reported that pre-lymphodepletion thrombocytopenia was not a risk factor associated with hematotoxicity after CAR-T therapy (26). Similarly, our results indicated that baseline blood cell counts were not significantly different between the BR and Non-BR subgroups, and none were correlated with early hematopoietic recovery (**Table 2**). Previous studies found that the CAR-T cell dose was closely associated with treatment response, and a higher CAR-T cell dose was an independent predictor of CRS (13, 29). However, the CAR-T cell dose was not a risk factor for hematopoietic recovery (19). In this study, we also observed that CAR-T cell dose was not significantly different between BR and Non-BR subgroups, and it was not a risk factor related to hematopoietic recovery. Recent studies have shown that baseline tumor burden notably increases the incidence of CRS, and high bone marrow tumor burden is an independent risk factor for CRS (17, 30). Moreover, Zhang’s research revealed that high bone-marrow blasts were an independent risk factor correlated with the CR rate in patients receiving CAR-T cell therapy (3). In B-ALL patients, we found that bone-marrow blasts were numerically higher in Non-BR patients than in BR patients, but the difference was not statistically significant. Meanwhile, the differences of Ann Arbor staging, IPI, and LDH levels between BR and Non-BR patients were not significant (**Table 2**, P > 0.05), the reason may be associated with the small sample size.

In addition, increased CRS severity was an independent factor affecting early BR. There was a significant difference in the severity of CRS between BR and Non-BR groups (**Table 2**). Moreover, our results indicated that the BR rate was lower in patients with grade ≥2 CRS, who had a worse OS than patients with a low CRS grade (**Supplementary Figure 1B**). Other studies have also demonstrated that a high CRS grade is an independent risk factor associated with early hematotoxicity (18, 19). Lastly, to construct an early predictive model of hematological toxicity, we traced the peak levels of various CRS-related cytokines during the first week following CAR-T treatment. These cytokines contained TNF-α, IL-10, IL-2R, IL-6, IL-8, and CRP. The results showed that the peak CRP levels were significantly higher in the Non-BR group than in the BR group. Meanwhile, lower peak levels of TNF-α were also observed in patients with BR than in those without BR, although the difference was not statistically significant (P = 0.07, **Figure 4A**). Other studies have also observed increased CRP levels in patients with early hematotoxicity (18, 24). In addition, Wang et al. found that TNF-α could produce an inhibitory effect on hematopoietic stem cells *via* the interleukin27Ra signaling pathway (31). Thus, it is reasonable to conclude that Non-BR patients had higher TNF-α levels. Combined with the results of discriminative ability and univariate analysis (**Figure 4**), TNF-α and CRP were identified as biomarkers to construct an early predictive model of hematotoxicity. In addition, we validated the predictive model in an independent cohort and the result suggested the the constructed model could early diagnose hematotoxicity well with AUC = 0.850. Nevertheless, this predictive model needs to be further validated in a larger prospective cohort because of the limited sample size in this study.

In conclusion, this study revealed that severe CRS was an independent risk factor for early hematopoietic recovery following CAR-T therapy. In addition, the peak levels of TNF-α and CRP during 1 week after CAR-T therapy were positively associated with early hematotoxicity. Using these data, we constructed an early predictive model of hematopoietic recovery based on the peak levels of TNF-α and CRP within 1 week following CAR-T therapy.

## 5 Study limitations

In this study, the severity of CRS was identified as an independent risk factor associated with early hematotoxicity and an early predictive model of hematotoxicity was constructed based on the peak levels of TNF-α and CRP. However, this study also had some limitations. Firstly, the number of patients was relatively small and our constructed predictive model needs to be further validated in a larger prospective study. In addition, most patients left hospital in one month after CAR-T therapy and we did not have long-term follow-up data of hematopoietic recovery. We will enroll more patients continuously and verify our results in the future study, meanwhile, we will pay more attention to the long-term follow-up data of hematopoietic recovery.

## Data availability statement

The original contributions presented in the study are included in the article/**Supplementary Material**. Further inquiries can be directed to the corresponding author/s.

## Ethics statement

The studies involving human participants were reviewed and approved by Institutional Review Board of Changhai Hospital. Written informed consent to participate in this study was provided by the participants’ legal guardian/next of kin.

## Author contributions

LW and JY were responsible for designing and guiding the study. YW and ZS analyzed the data, made the figures, and wrote the manuscript. YW, YG, LG, LX, JC, TW, and WF collected the data and provided clinical consultation. YG, GT, XN, and LC made the tables and revised manuscript. DF and XY provided CAR-T cells and performed the analysis of CAR-T cells. LW and JY revised the manuscript. All authors contributed to the article and approved the submitted version.

## Funding

YW received funding from Youth Start-up Fundation of the First Affiliated Hospital of the Second Military Medical University. LW received funding from the Natural Science Foundation of Shanghai (20ZR1457100). JY received funding from the National Natural Science Foundation of China (NSFC) (81770209) and Shanghai 2021 “Action Plan of Technological Innovation” Biomedical Science and Technology Support Special Project (21S11906100).

## Conflict of interest

Authors DF and XY are employed by HuaDao Biopharma (Shanghai) Limited Corporation, a biotechnology company focusing on the research and development of cellular immunotherapy.

The remaining authors declare that the research was conducted in the absence of any commercial or financial relationships that could be construed as a potential conflict of interest.

## Publisher’s note

All claims expressed in this article are solely those of the authors and do not necessarily represent those of their affiliated organizations, or those of the publisher, the editors and the reviewers. Any product that may be evaluated in this article, or claim that may be made by its manufacturer, is not guaranteed or endorsed by the publisher.
