# Supplementary material for: The risk factors and early predictive model of hematotoxicity after CD19 chimeric antigen receptor T cell therapy
Source: Front Oncol. 2022 Sep 30;12:987965. doi: 10.3389/fonc.2022.987965 (PMC9561932; doi:10.3389/fonc.2022.987965)
Supplement: Supplementary file 1 [file DataSheet_1.docx]

The risk factors and early predictive model of hematotoxicity after CD19 chimeric antigen receptor T cell therapy

**Supplementary materials:**


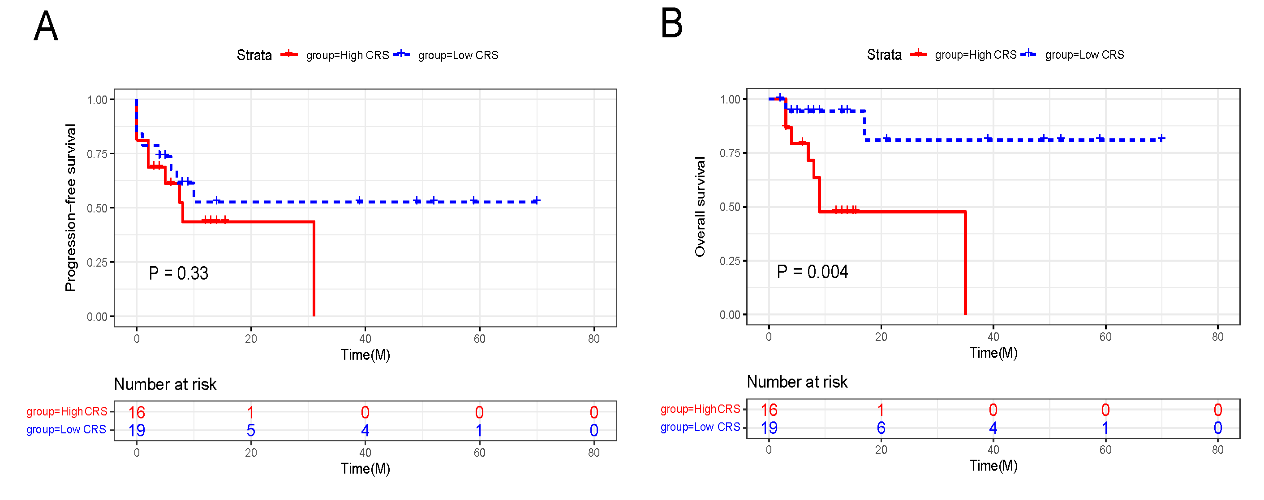


**Supplementary Figure 1.** The effects of severity of CRS on PFS (A) and OS (B) probability. The survival analysis revealed that PFS had no significant difference between patients with low and high CRS grade while patients with low CRS grade had longer OS in the training cohort. CRS, cytokine release syndrome; PFS, progression-free survival; OS, overall survival.

**Supplementary Table S1.** Factors affecting BR by multivariate logistic regression analysis.

| Variables | P value | | OR | 95%CI | |
| --- | --- | --- | --- | --- | --- |
|  |  |  |  | Lower | Higher |
| Baseline WBC | | 0.572 | 0.954 | 0.812 | 1.122 |
| Baseline hemoglobin | | 0.326 | 0.979 | 0.940 | 1.021 |
| Baseline platelet | | 0.098 | 0.988 | 0.973 | 1.002 |
| CRS grade (≥2 vs. <2) | | 0.038 | 7.117 | 1.112 | 45.546 |

BR, blood complete recovery; OR, odds ratio; WBC, white blood cell; CRS, cytokine release syndrome.
